# THz Radiation Efficiency Enhancement from Metal–ITO Nonlinear Metasurfaces

**DOI:** 10.1021/acsphotonics.2c01447

**Published:** 2022-12-01

**Authors:** Symeon Sideris, Eviatar Minerbi, Cormac McDonnell, Tal Ellenbogen

**Affiliations:** † Department of Physical Electronics, School of Electrical Engineering, Tel-Aviv University, Tel Aviv6997801, Israel; ‡ Center for Light−Matter Interaction, Tel-Aviv University, Tel Aviv6779801, Israel; § Raymond and Beverly Sackler Faculty of Exact Sciences, School of Physics & Astronomy, Tel-Aviv University, Tel Aviv6779801, Israel

**Keywords:** optical rectification, indium tin oxide (ITO), terahertz emission, radiation efficiency, metasurface

## Abstract

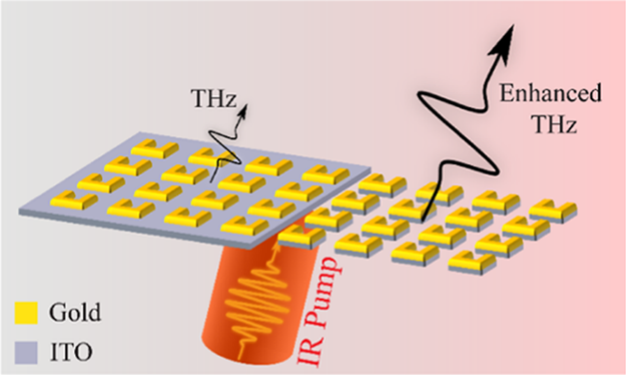

Strong single-cycle THz emission has been demonstrated from nonlinear plasmonic metasurfaces, when excited by femtosecond laser pulses. In order to invoke a higher nonlinear response, such metasurfaces have been coupled to thin indium-tin-oxide (ITO) films, which exhibit an epsilon-near zero (ENZ) behavior in the excitation wavelength range and enhance the nonlinear conversion. However, the THz conductivity of the ITO film also reduces the radiation efficiency of the meta-atoms constituting the metasurface. To overcome this, we etch the ITO layer around the plasmonic meta-atoms, which allows harnessing of the enhanced localized fields due to the ENZ behavior of the remaining ITO film, while improving the THz radiation efficiency. We report an increase of more than 1 order of magnitude in the emitted THz spectral power density, while the energy conversion efficiency approaches 10^–6^. This simple yet very effective fabrication scheme provides important progress toward increasing the range of applications of nonlinear plasmonic metasurface THz emitters.

## Introduction

The terahertz (THz) electromagnetic spectral region has been a topic of increased interest over the past few years.^1^ These radiated waves, which correspond to frequencies in the range of 0.1–10 THz, have been highly suitable for numerous applications, such as nondestructive material evaluation,^2,3^ biomedical imaging,^4,5^ and high-speed wireless communication systems.^6,7^ So far, a plethora of sources has been developed for the generation and shaping of THz radiation, including photoconductive antennas,^8^ nonlinear crystals,^9^ and spintronic emitters,^10^ among others. Recently, a novel set of ultra-thin emitters based on nonlinear plasmonic metasurfaces has been developed, demonstrating complete spatio-temporal control of the emitted THz radiation^11−15^ by exploiting the ability to spatially tailor the arrangement of meta-atoms constituting the nonlinear metasurface (NLMS). Interestingly, this platform provides conversion efficiencies similar to much thicker conventional ZnTe electro-optic crystals,^11,16^ highlighting its importance on the THz generation.

While investigating the origin of high conversion efficiency of NLMSs on THz generation, it was lately revealed that the enhanced nonlinear activity is associated with a thin indium-tin oxide (ITO) film,^15,17^ on top of which the NLMSs are fabricated. Fundamentally, the large nonlinear response of ITO films has been attributed to the enhancement of the normal component of the electric field at the epsilon-near-zero (ENZ) region.^18−20^ To exploit this large nonlinearity under normal incidence illumination, NLMSs have been coupled to thin ITO layers,^15,21−23^ exhibiting increased second- and third-order nonlinearities. Moreover, the hot-electron dynamics present in the ITO–NLMS system strongly modify the coupling in the sub-picosecond time scale, which results in the dynamic control of the bandwidth of the emitted THz signal.^17^ These findings cement the ITO film as an important component in the design of NLMSs for the generation and shaping of THz radiation. However, the THz conductivity of the thin ITO film may also dampen the free space radiation. In this work, we focus our work on enhancing the THz emission from NLMSs using a simple and inexpensive fabrication method which incorporates nanostructuring of the ITO layer.

## Results and Discussion

### Dipole Approximation

Considering a metasurface consisting of split ring resonators (SRRs) on top of an ITO-coated glass, after illumination with an ultra-short pulse in the vicinity of its magnetic dipole resonance, strong nonlinear currents form on the meta-atoms and the ITO.^24−28^ Due to the scale of the meta-atoms, which is a 1000 times smaller than the THz wavelength, the nonlinear currents turn the nanoscale meta-atoms into Hertzian dipoles emitting THz waves. However, owing to the metallic behavior of the ITO in the THz regime,^29,30^ the radiation efficiency of the infinitesimal dipoles is depleted.^31^

**Figure 1:**
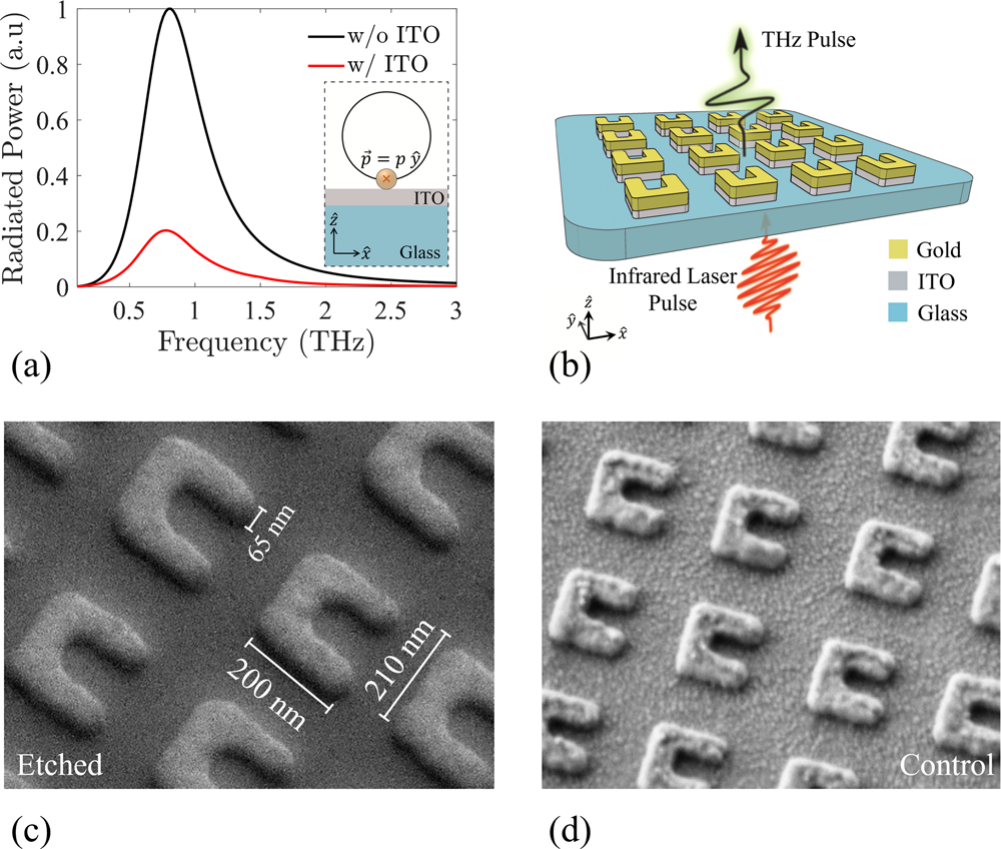
(a) Radiated power emitted from a square lattice of point dipoles with a period of 380 nm, and polarizability **p** along *ŷ*. In the absence of the 20 nm thick ITO (black curve), the radiated power is 5 times higher than with it (red curve). The inset illustrates the point dipole approximation we used in our simulations. (b) Illustration of our proposed metasurface scheme for the enhanced THz generation from NLMSs. The metasurface consists of an alternating layer of gold and ITO split ring resonators (SRRs). (c) Variable pressure scanning electron microscopy (SEM) imaging of the sample following the etching of the ITO layer. The arms and base of the SRRs are measured as 200 and 210 nm long, respectively, with a width of 65 nm. The sample was imaged at a 10°-tilt stage. (d) SEM image of the control sample. The dimensions of the meta-atoms are identical to those of the etched sample. The imaging angle is 22°.

By modeling the SRRs as point dipoles in a square lattice with a period of 380 nm, the effect of the 20 nm thick ITO film on the emitted THz waves was investigated by simulating two lower-half space cases. In the first case, the lower half-space consisted of ITO-coated glass (see inset of Figure 1a), while the second consisted of bare glass. The polarizability (**p**) of the dipoles followed a Lorentzian shape along *ŷ*, according to , where *f*, *f*_0_, and Γ denote the frequency, central frequency, and full width at half maximum, respectively. Following a fit to previous experimental data,^11^*f*_0_ and Γ were set to 0.7 and 0.8 THz, accordingly. Periodic boundary conditions were applied on all sides and absorbing boundary conditions were used to terminate the computational domain along *ẑ*. Assuming the dipole polarizability remained unaltered for both cases under the study, we get a direct estimation of the radiation efficiency of the Hertzian dipole arrays, which is independent of the meta-atom’s shape. According to the simulation results, in the absence of the thin ITO layer, the metasurface emits THz waves 5 times more efficiently on the air side (Figure 1a). In order to increase the radiation efficiency of the THz emitters, while simultaneously harnessing optimally the local field enhancement in the ENZ region of the ITO layer, we propose a metasurface scheme where the ITO surrounding meta-atoms is etched away, as illustrated in Figure 1b.

### Linear Characterization

To verify this claim experimentally, two 1 mm × 1 mm metasurfaces of identical SRR unit cells were fabricated, as presented on Figure 1c,d. The ITO surrounding the SRRs was completely removed through reactive ion etching (RIE) on the first sample, whereas on the second sample the ITO was left untreated, which served as the control case. The fabrication steps are presented in detail in the Supporting Information (Section S1). Following the fabrication, the samples were imaged by a scanning electron microscope (Figure 1c,d). After removing the ITO layer, the etched sample was imaged in variable pressure scanning electron microscopy (SEM) due to its lack of conductivity. The differences in the surface morphology of the SRRs in the two samples are attributed to the lower imaging resolution of the variable pressure SEM. The grainy texture of the substrate before etching is due to the presence of the ITO film.

**Figure 2:**
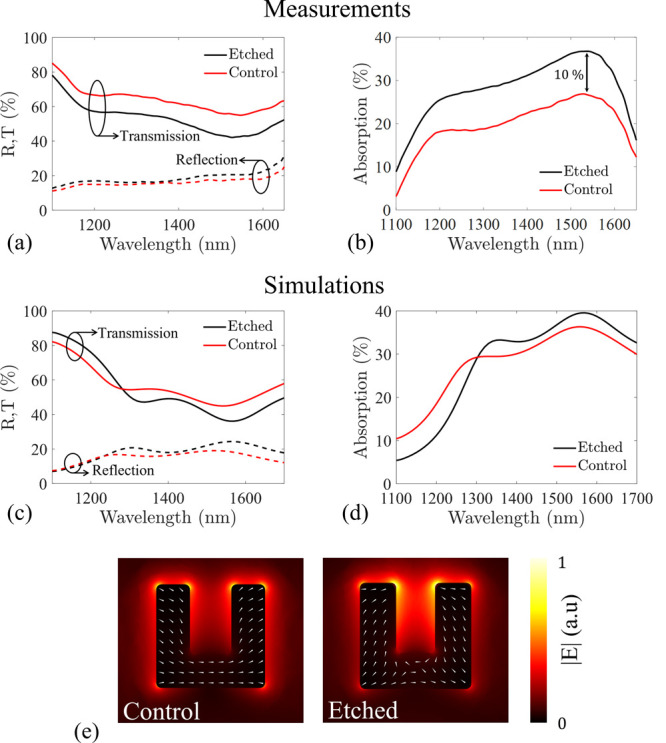
(a) Linear transmission (*T*—continuous lines) and reflection (*R*—dashed lines) measurements. The black curves refer to the etched sample and the red ones to the control. Both samples show transmission dips at 1200 and 1550 nm, indicating the existence of localized surface plasmon resonance. (b) Absorption measurements of the etched (black) and control (red) samples. The etched sample absorbs 10% more power throughout the resonant bandwidth. (c) Finite element simulation of the etched (black) and control (red) metasurfaces. The etched metasurface shows transmission dips at 1300 and 1570 nm and resonant behavior similar to the control, which exhibits transmission dips at 1250 and 1550 nm. (d) Absorption calculation of the etched (black) and control (red) metasurfaces. (e) Electric field distributions of the simulated nanoparticles at 1550 nm excitation. Both resonators exhibit similar modal distributions, with 1.3-times higher field localization in the etched metasurface. The simulated profiles share the same color bar.

To obtain the linear optical properties of our samples, a transmission–reflection setup was used. The metasurfaces were excited with linearly polarized light along the base of the SRRs (*x̂*-direction) and an identical localized surface plasmon resonant behavior was observed as transmission dips between 1150 and 1650 nm (Figure 2a), which correspond to the magnetic dipole mode. The transmission levels of the etched sample were ∼10% lower than the control. The transmission through each substrate was used as the reference measurement for both metasurfaces, and a flip-mirror was used as a reference for the reflection measurements. A schematic of the linear characterization setup can be found in Section S2 of the Supporting Information. The reflection measurements of the samples did not display any significant difference, and we did not observe pronounced scattering; therefore, the etched sample shows higher absorption levels all over the resonant wavelength range, as shown in Figure 2b. The absorption (*A*) of the samples is calculated according to *A* = 1 – *T* – *R*. An increase in the absorption suggests higher Ohmic losses and increased localized fields, which can potentially lead to a stronger nonlinear response as previous studies have reported.^32−35^

The linear response of the two samples was simulated using the finite element method with periodic boundary conditions applied on all sides. The extracted response is presented in Figure 2c,d, showing resonant behavior in the bandwidth between 1250 and 1650 nm. Initial simulation results predicted a redshift in the resonance of the etched sample, relative to the control (Figure S1). In order to fit the linear characteristics of the etched metasurface, 2.5 nm of a thin SiC film were applied to the sides of the simulated meta-atoms. We speculate that such residues were potentially deposited during the RIE process on the sidewalls of the SRRs,^36^ due to the exposure of SiO_2_ to a CHF_3_ reactive ion plasma.^37^ The near field distributions assuming excitation at 1550 nm are presented in Figure 2e, which show an increase in the intensity of the local fields in the etched metasurface. A numerical estimation of the local field revealed ∼1.3 times stronger near field distribution in the etched configuration, indicating ∼3 times higher second-order nonlinear intensities. The detailed characterization of the excited modes can be found in the Supporting Information (Section S3).

### Enhanced Nonlinear Emission

**Figure 3:**
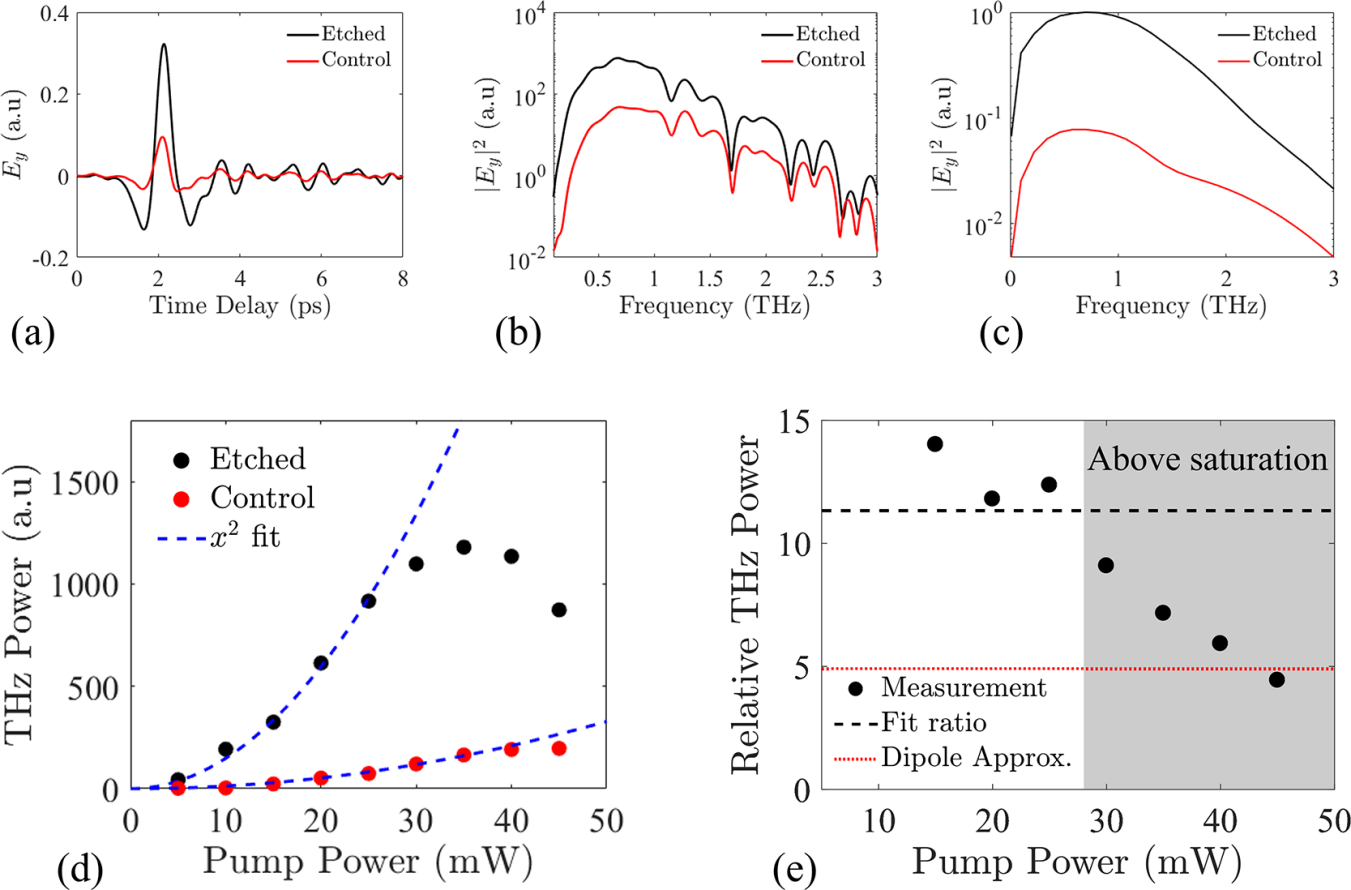
(a) Single cycle pulse emission of the etched (black) and control (red) NLMSs under 25 mW incident power. The relative peak to peak ratio shows 3.4 times enhanced emission in the etched sample. (b) Power spectral component of the pulses measured in (a). Both samples emitted a spectral bandwidth of 3 THz, with the etched sample showing 12 times increased power. (c) Nonlinear simulation of the THz emission of the etched (black) and control (red) NLMSs. The hydrodynamic model predicts 15 times higher THz emission for the etched sample, with identical spectral components to the control. (d) Power measurements of the etched (black) and control (red). The samples showed quadratic behavior (dashed) until saturation was reached. (e) Power comparison between the samples. The etched sample showed enhanced THz emission, with up to 14 times higher power spectral components. An estimation of the enhancement achieved in this work is given by the fit ratio (dashed) of the power measurements in (d), resulting in 11 times increased emission. The dipole approximation (dotted) yielded around 5 times enhancement, as it does not capture the effect of absorption of the nanoparticles. Above saturation (gray area), the enhancement follows a descending trend as we approach the damage threshold of the NLMSs.

Continuing our assessment, we examined the THz waves emitted from our samples using a standard THz time-domain spectroscopy (TDS) setup. Each sample was illuminated with *x̂*-polarized ultrashort laser pulses (∼50 fs) with 2 kHz repetition rate. All wavelengths over the resonant range can be used to generate THz emission.^15^ In our experiment, a 1550 nm central wavelength was incident from the glass side, to excite the magnetic resonant mode. The THz emission was collected from the air side of the sample (Figure 1a), with a parabolic mirror (*f* = 101.6 mm) and after collimation, it was electro-optically sampled. The complete experimental setup is presented in the Supporting Information (Section S4). Throughout the measurements, both samples emitted THz waves polarized along the arms of the SRRs (*ŷ*-direction). As seen in Figure 3a, both samples emitted similar single cycle pulses of ∼1 ps duration, as reported in previous works.^11,16^ At an incident power of 25 mW, corresponding to a fluence of 176 μJ/cm^2^, the etched sample emitted a THz field with a peak-to-peak amplitude 3.4 times higher than the control. The spectral component of these measurements spanning over 3 THz is shown in Figure 3b, which reveals a remarkable 12 time enhancement in the emitted THz power. These results are additionally supported by simulations based on the hydrodynamic model of the electron motion responsible for the nonlinear THz emission.^17,27^ In agreement with the experimental measurements, the nonlinear simulations predict 15-fold enhancement in the emitted THz power due to the combined effect of increased radiation efficiency and field localization (Figure 3c). An in-depth description of the simulation details is presented in Section S5 of the Supporting Information.

The dependency of the THz intensity on the pumping power of the samples is presented in Figure 3d. At first, it can be observed that the etched sample generated THz waves of higher intensities throughout the experimental range. Additionally, both samples expressed a quadratic behavior relative to the pumping power. As stated in previous works, this suggests a second-order nonlinear process, namely optical rectification, as the generation mechanism of the THz waves.^16,27,38−40^ After the quadratic increase in the THz power curves in Figure 3d, a reversible saturation behavior is observed, which relates to the hot-electron dynamics in the system.^17^ The saturation point in the generated THz intensity of the etched sample is around 30 mW, which is lower than the control’s (40 mW). The shift of the saturation point is attributed to the higher absorption of the nanoparticles in the etched configuration. Moreover, the maximum THz intensity of the control sample (at 40 mW pumping power) can be attained using only 10 mW on the proposed sample. This is a very advantageous feature on the enhancement of THz emission from NLMSs because it allows the excitation of samples with 4 times lower pumping power, thus significantly lowering the risk of sample damage, which is a common issue with plasmonic metasurfaces.

To get a qualitative idea of the enhancement attained by etching the ITO layer surrounding the metasurface, the relative THz intensity of the etched sample is displayed in Figure 3e. At pumping powers up to 10 mW, the control sample did not emit detectable signals. To compensate for the low signals emitted from the control, a conservative estimation was used as the quadratic fit ratio of the power measurements in Figure 3d. The fit ratio revealed 11 times enhancement in the emitted THz powers and is a valid estimation up to the first saturation point. In the region from 15 mW up to the saturation point of the etched sample (30 mW), our approach yielded more than an order of magnitude stronger THz emission. According to the dipole approximation, a 5 time enhancement was expected. The extra increase of about 2.5 times in our experimental data is credited to the enhanced local fields on the meta-atoms, following the etching process of the ITO layer. Finally, above the saturation point of the etched metasurface the enhancement decreases because the control sample has an increasing power trend until it saturates at ∼40 mW. Importantly, as discussed in our previous work, at such high pumping powers, the thermo-optical changes in the permittivity of the ITO enable the pulse shortening, thus the spectral broadening of the emitted THz waves.^17^ This dynamic behavior of the ITO layer is present in our control sample, in comparison to the etched one (Figure S5a,b). This shows that the dynamic broadening of the THz waves is due to electron and lattice heating of the surrounding ITO film. Thus, by removing it, the effective thermo-optic response of the system becomes smaller. At this point, a rough estimation of the conversion efficiency of the NLMSs will give us physical insight on the strength of the THz emission. According to preceding experimental data, SRRs have shown conversion efficiencies that are comparable to 2000 times thicker ZnTe crystals.^11^ These results can be used to estimate their conversion efficiency to be around 9 × 10^–8^.^11,41^ Therefore, the estimated conversion efficiency of the etched metasurface at the excitation conditions that we used is calculated as 1 × 10^–6^, bridging the gap between NLMSs, thick inorganic crystals, and also photoconductive antennas^42^ in the generation of THz waves.

**Figure 4:**
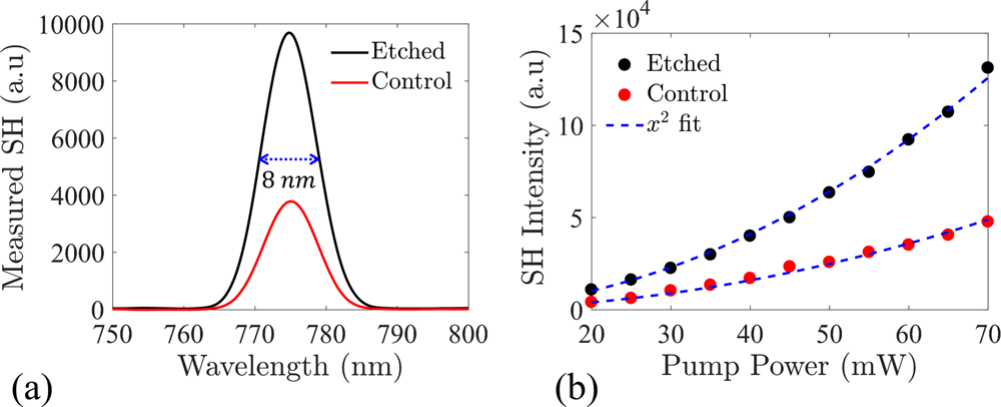
(a) Measured second harmonic spectra, after illumination with 60 mW. Both samples exhibit 8 nm linewidth with a spectral peak located at 775 nm. The etched sample (black) emitted 2.6 stronger SH. (b) Power characterization of the emitted SH. The quadratic fit ratio of the data reveals 2.6 times enhanced SH emission, due to the increased absorption of the etched sample.

Finally, to assess the sole effect of the increased absorption in another second-order nonlinear process, we measured the second harmonic (SH) generated from our samples. Our samples were illuminated at 1550 nm with 80 MHz repetition rate, and we measured the generated SH from the air side of the samples (Figure S6). As we observe in Figure 4a, both samples emitted strong SH signals centered at 775 nm with a 8 nm linewidth. In our power measurements (Figure 4b), both samples show a quadratic behavior which was expected for second-order processes. The etched sample emitted stronger SH signals, with the fit ratio of the experimental curves showing 2.6 times enhancement in the SH emission. Consequently, the enhancement in the SH emitted from our etched sample is a byproduct of the higher localized fields on the meta-atoms, in close agreement with the theoretical prediction, and a potential modification of the effective χ^(2)^ of the metasurface.

## Conclusions

In conclusion, we show that the conductive nature of the ITO film in nonlinear THz emitting metal–ITO metasurfaces depletes the generated radiation. To overcome this problem, we propose a fabrication scheme which allows harnessing of the strong nonlinearities provided by the ENZ response of the ITO, without paying the price of reduced THz radiation efficiency. To achieve this, we etch the ITO film surrounding the meta-atoms and obtain up to 14 times enhanced THz emission, compared to a metasurface fabricated on top of an ITO-coated glass. Additional improvement on the radiation efficiency may be realized by optimally shaping the structural properties of the metal–ITO nanoinclusions. Nonetheless, considering the simplicity in the fabrication steps of our proposed scheme, we believe our work will encourage further radiation optimization schemes and pave the way towards more efficient, compact, and fully integrated sources for the generation and shaping of THz waves.

## Supporting Information Available

Sample fabrication, linear characterization setup, modal analysis of the resonators, THz TDS spectroscopy system, nonlinear hydrodynamic model, THz emission spectra, and SH measurement setup (PDF)

## Notes

Data Availability: The data that support the findings of this study are available from the corresponding author upon reasonable request.
