# Supplementary material for: THz Radiation Efficiency Enhancement from Metal–ITO Nonlinear Metasurfaces
Source: ACS Photonics. 2022 Dec 1;9(12):3981–6. doi: 10.1021/acsphotonics.2c01447 (PMC9782780; doi:10.1021/acsphotonics.2c01447)
Supplement: Supplementary file 1 — ph2c01447_si_001.pdf [file ph2c01447_si_001.pdf]

Supplementary Information

THz Radiation Efficiency Enhancement from Metal-ITO Nonlinear Metasurfaces

Symeon Sideris[1,2,*], Eviatar Minerbi[2,3], Cormac McDonnell[1,2] and Tal Ellenbogen[1,2]

[1]*Department of Physical Electronics, School of Electrical Engineering, Tel-Aviv University, Israel*
[2]*Center for Light-Matter Interaction, Tel-Aviv University, Israel*
[3]*Raymond and Beverly Sackler Faculty of Exact Sciences, School of Physics & Astronomy, Tel-Aviv University, Israel*

**Corresponding author: symeons@mail.tau.ac.il*

Supplementary Material

S1. Sample Fabrication

The metasurfaces (named Etched and Control in the main text) used in this study were fabricated on a commercial ITO-coated BK7 glass, with a sheet resistance of 70-100 Ω/sq. Initially, the substrates were cleaned by sonication in acetone and dried under a stream of N_2. After the cleaning process, polymethyl methacrylate (PMMA A4) was spin-coated on the substrates, which were later baked at 180 $°C$ on a hot plate for 1 min. The metasurfaces were written using a standard electron beam lithography system (Raith 150 – II) at 20 kV. To develop the patterns, the substrates were immersed in a MIBK/IPA 1:3 solution for 1 min, and dried under a dry stream of N_2. Afterwards, 3 nm of Ti were evaporated, followed by 37 nm of gold and 25 nm of Cr. The remaining photoresist was lifted-off in acetone.

The ITO layer was afterwards removed from one metasurface using reactive ion etching (RIE). The RIE process was performed on a Oerlikon 790 Reactive Ion Etcher, with a gas mixture consisting of CHF3 and Ar. The gas flow rates were set to 40 ccm for CHF3 and 10 ccm for Ar, and the gas mixture was excited with 250 W pumping power, while the chamber's pressure was kept at 40 mTorr. The sample was etched for 3 mins.

In order to remove the Cr layer, the samples were immersed in a commercial Cr etchant solution for 1 min, followed by another immersion in deionized water for 1 min and dried under a stream of N_2.

We note that due to the lack of conductivity, the etched sample was imaged in a variable pressure SEM chamber.

S2. Linear Characterization Setup

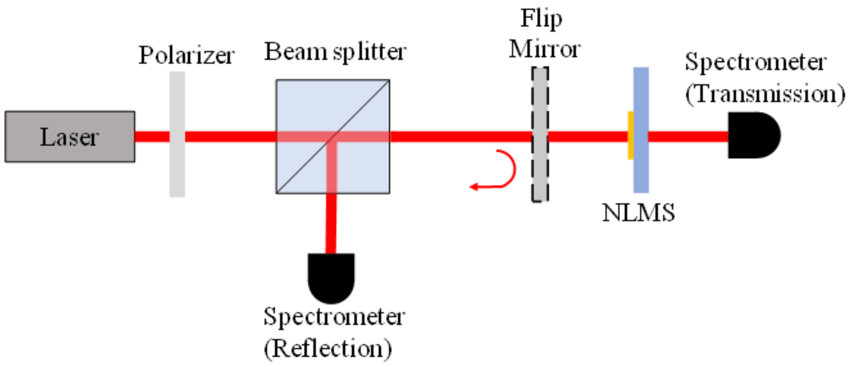

Figure S1. Schematic illustration of the linear characterization setup.

S3. Supplementary Linear Simulations

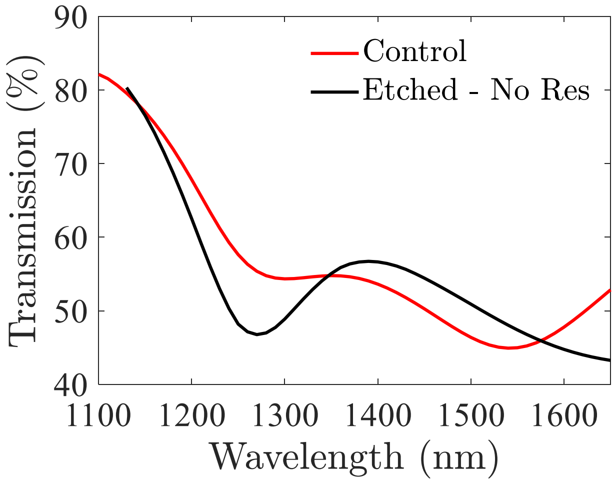

Figure S2. Transmission of the etched metasurface without applying SiC residues on the sidewalls.

In Fig. S3 we present the linear mode profiles assuming excitation at 1550 nm for the control, and etched (with and without SiC residues deposited on the sidewalls). As we see, both modes are better confined in the the etched samples, due to the discontinuity of the ITO. In the presence of the SiC residues, the arms of the meta-atoms are showing better coupling due to the presence of the high-index dielectric film on them. All 3 cases exhibit the magnetic dipole mode, as depicted in the volume field distribution profiles and the arrow distributions on the surfaces of the resonators. According to our calculations, the local field enhancement is calculated as ~1.3 and ~1.25 for the etched samples with and without residues respectively, relative to the control, indicating stronger nonlinear response.

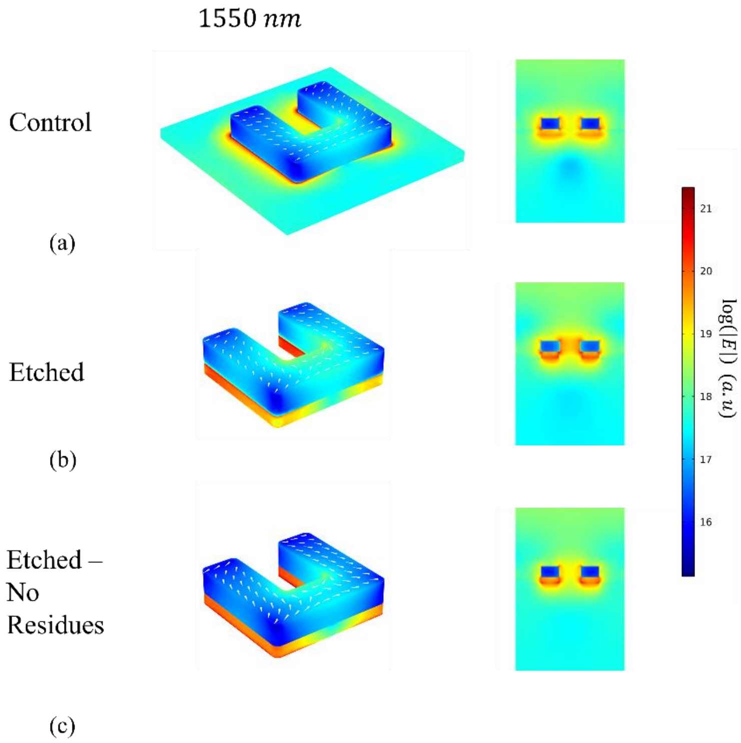

Figure S3. Simulated near field profiles of the (a) Control, (b) Etched with SiC residues and (c) Etched without SiC residues metasurfaces.

S4. Terahertz Time Domain Spectroscopy

To measure the THz waves emitted from the fabricated metasurfaces, a time domain spectroscopy (TDS) setup was employed, as presented in Fig. S4. A femtosecond laser source was used, emitting pulses of ~50 fs duration (Spectra-Physics Solstice Ace) in a spot-size of 3 mm diameter, with 2 kHz repetition rate and 3.5 mJ pulse energy. The laser output is split into two optical lines, namely pump and probe, with power ratio 99:1.

The pump line is directed into an optical parametric amplifier (TOPAS), allowing the generation of ultra-short pulses at 1550 nm that was used to excite the metasurfaces, followed by a chopper at 1 kHz. The THz waves emitted from the metasurfaces were collected and collimated by an off-axis parabolic mirror with focal length $f = 101.6$ mm, and a Teflon slab was adopted to filter the pump line after the excitation of the samples. The THz waves were then focused on a 0.5 mm ZnTe crystal with another off-axis parabolic mirror with $f = 101.6$ mm.

In the probe line, the light beam is directed in a motorized delay stage, which controls the temporal overlap between the probe and the THz pulses on the ZnTe electro-optical crystal. As the THz signal induces birefringence in the ZnTe crystal, the probe signal's polarization is rotated. To detect this modification in the polarization, the probe then passes through a quarter waveplate, a Wollaston prism and finally a balanced photodetector. The photodetector is connected to a lock-in amplifier (Stanford Research Systems SR830), which is synchronized with the chopper, to increase the SNR of the system. The setup was automated using LabView.

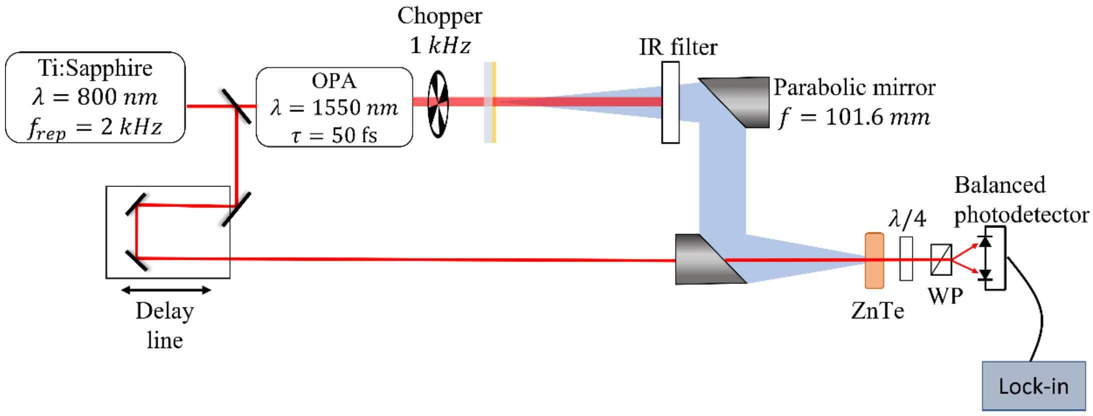

Figure S4. Schematic representation of the THz – TDS setup.

S5. Hydrodynamic Model – Nonlinear THz Emission Simulation

Following our previous works,[1,2] we described the nonlinear response of the electrons in the gold and ITO layers according to a Maxwell – Hydrodynamic model. This model treats the electron motion according to the Euler's equation:

$$\frac{\partial \boldsymbol{v}}{\partial t} + (\boldsymbol{v} \cdot \nabla)\boldsymbol{v} + \Gamma \boldsymbol{v} = \frac{e}{m^*}(\boldsymbol{E} + \boldsymbol{v} \times \boldsymbol{B}) - \frac{\beta^2}{N}\nabla N \qquad (5.1)$$

where $\boldsymbol{v}$ and N are the electron velocity and density, $\boldsymbol{E}$ and $\boldsymbol{B}$ are the electric and magnetic field distributions, e and m^* are the electron charge and effective mass respectively and β is related to the electron pressure. After expanding perturbatively Eq. 5.1 and applying the proper continuity conditions, we can describe the difference frequency generation from NLMSs as an equivalent nonlinear surface current density.

This current density reads:

$$\boldsymbol{K}_{NL} = -\frac{i}{eN_0}\Big[\hat{\boldsymbol{t}}\left(\omega_1 P_2^{\perp *} P_2^{\parallel} - \omega_2 P_1^{\perp} P_2^{\parallel *}\right) + \hat{\boldsymbol{n}}\left(\frac{1}{2}\omega_3 - \frac{\omega_1\omega_2}{\omega_3 + i\gamma} P_1^{\perp} P_2^{\perp *}\right]\qquad (5.2)$$

where $\boldsymbol{P}_{1,2}$ denotes the polarization vector induced after excitation at frequencies ω_1, ω_2.This current density is applied on the gold nanoparticle and the ITO. The simulation is performed in a 3-step process, where frequencies ω_1 and ω_2 simulate the linear excitation of the sample, and the frequency $\omega_3 = \omega_1 - \omega_2$ corresponds to the nonlinear term with the surface current as the exciting source of THz emission. Periodic boundary conditions are applied on the sides of the domain and absorbing boundary conditions on the direction of propagation.

S6. Terahertz Emission Spectra

The emitted THz spectra of the metasurfaces are shown in Fig. S5. The spectral components of the etched sample did not show any significant dependency on the excitation power (Fig. S5 (a)), in contrast to the control sample (Fig. S5 (b)).

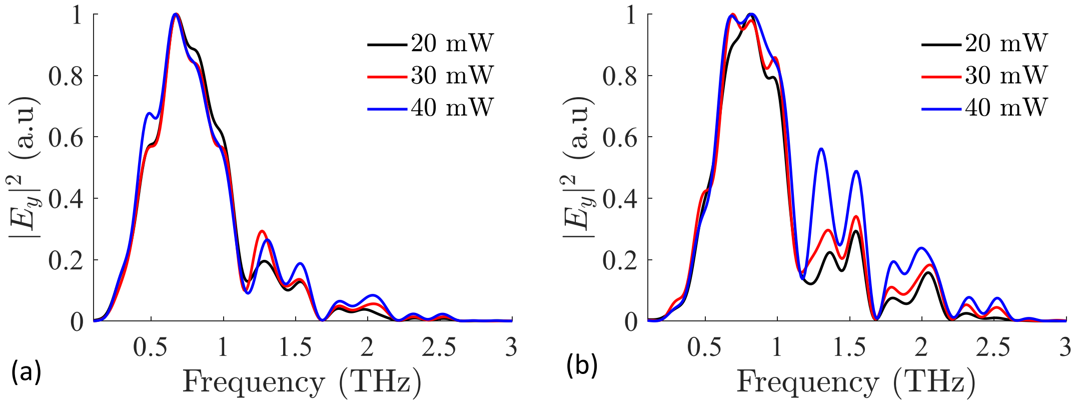

Figure S5. THz emission spectra of the Etched (a) and Control (b). The Control sample's emission spectrum shows a dependency on the excitation's power, in contract to the Etched.

S7. Second Harmonic Measurement Setup

The second harmonic emitted from the NLMSs was measured using the setup shown in Fig. S6. An Optical Parametric Oscillator (OPO) was used to excite the samples, emitting pulses of 170 fs duration with a repetition rate of 80 MHz. The metasurfaces were placed at the focal point of a lens (f = 100 mm) and the second harmonic was collected from the air side, using an objective lens (20 X). The second harmonic was then split into two paths, one leading to the spectrometer and the other to a CCD camera.

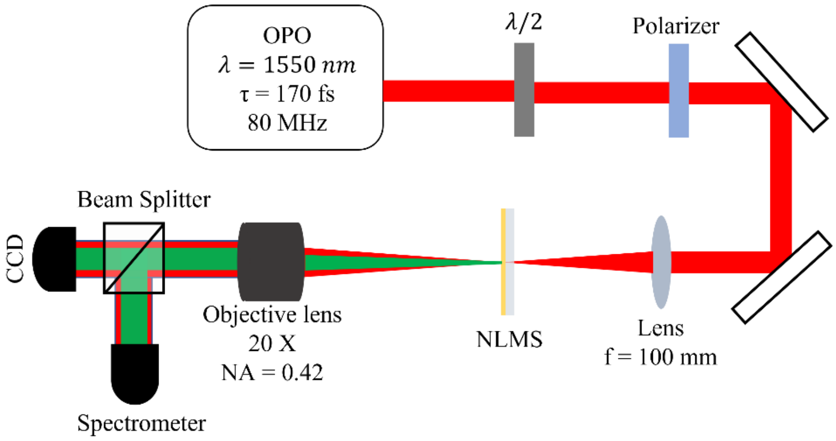

Fig. S6. Schematic representation of the second harmonic measurement setup. The emitted second harmonic (green beam) is collected from the air-side of the sample.

References

(1) Sideris, S.; Ellenbogen, T. Terahertz Generation in Parallel Plate Waveguides Activated by Nonlinear Metasurfaces. *Opt. Lett.* **2019**, *44* (14), 3590. https://doi.org/10.1364/OL.44.003590.

(2) Minerbi, E.; Sideris, S.; Khurgin, J. B.; Ellenbogen, T. The Role of Epsilon Near Zero and Hot Electrons in Enhanced Dynamic THz Emission from Nonlinear Metasurfaces. *Nano Lett.* **2022**, *22* (15), 6194–6199. https://doi.org/10.1021/acs.nanolett.2c01400.
